# Parental Involvement in Decision‐Making About Planned Late Preterm and Early Term Birth (The “PIP” Study): Part 1—A Reflexive Thematic Analysis of Interviews With Parents

**DOI:** 10.1111/birt.70025

**Published:** 2025-10-31

**Authors:** Frances J. Mielewczyk, Caroline A. Mulvaney, Elaine M. Boyle

**Affiliations:** ^1^ School of Healthcare University of Leicester Leicester UK

**Keywords:** decision‐making, early term, late preterm, parental involvement, pregnancy, qualitative, reflexive thematic analysis

## Abstract

**Introduction:**

Late preterm and early term birth are associated with increased risks of adverse health outcomes throughout life. Where such births have been decided upon in advance, many parents are dissatisfied with the input they are able to have in the decision‐making process. This paper reports a qualitative investigation exploring the input parents want and expect in decision‐making about possible planned LPET birth; how their experiences compare with these; and how they feel about the input they are able to have.

**Method:**

Semi‐structured interviews were conducted with parents of babies up to 6 months old, who had been involved in discussions with doctors about the possibility of planned late preterm or early term birth. Data were analyzed using Reflexive Thematic Analysis.

**Results:**

Twelve parents of nine singleton babies took part. Analysis generated three themes: (1) *What matters most to us in this decision?* (2) *We are in a new and strange place*, and (3) *Can we work together to reach a decision?* Each theme encompassed two or more sub‐themes.

**Conclusion:**

Parents want to feel able to voice their questions and concerns; to understand why early birth is being considered, their options and the reason behind specific recommendations; and to work with healthcare professionals to reach a plan that is agreed by all as best for the baby, inclusive of other issues they consider important, and carried out as planned. Suggestions are made for what parents need if these wishes and expectations are to be met.

## Graphical abstract

Parents involved in discussions about possible planned late preterm and early term (LPET) birth are often dissatisfied with the input they are able to have. In this qualitative investigation, interviews were held with parents of babies younger than 6 months old, who had been involved in discussions with doctors about the possibility of planned LPET birth. Twelve parents of nine singleton babies took part (9 mothers and 3 fathers). Results showed that parents want to feel able to: have a voice, understand their options and the reasons behind recommendations, and work with healthcare professionals to reach an agreed plan.

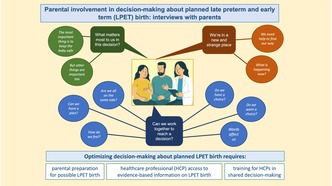

## 1 Background

Around 30% of live births in England and Wales (over 200,000 per year) occur late preterm (34^+0^ to 36^+6^ weeks of gestation) or early term (37^+0^ to 38^+6^ weeks) [1]. While many babies born at these stages do well, compared to those born full‐term they are at increased risk of neonatal morbidities [2, 3, 4] and ongoing health issues that may persist throughout life [5, 6, 7, 8, 9, 10, 11, 12]. In addition to the impact on affected individuals and families, the large numbers involved place significant demands on healthcare resources [13].

While many late preterm and early term (LPET) births occur spontaneously, others reflect decisions in which the risks of continuing a pregnancy have been judged to outweigh those of early birth. Although little is known about the processes involved in reaching such decisions or about parental involvement, it has been found that most expectant parents want to contribute to obstetric decision‐making [14, 15, 16, 17] and their inclusion is now widely recommended [18, 19, 20, 21, 22, 23, 24]. However, parents continue to feel dissatisfied with their involvement, with some men feeling excluded from the process [14, 25] and up to 51% of women viewing their input as too little [15, 16, 17]. Power inequalities between parents and clinicians during decision‐making have been identified as important in this, with the balance of power being particularly affected by clinicians' opinions about parents' right to have their preferences taken into account versus their own responsibility for the safety of mothers and babies [18, 22, 26, 27]. Other influences on the extent to which parents have input to the decision‐making process include contextual factors such as the presence or absence of medical indicators for a particular procedure and the strength of the associated evidence‐base [26, 28, 29], the quality of information provision and communication [20, 22, 30], and both demographic and other individual characteristics [15, 27, 31].

A recent literature review [32] highlighted the need to explore how decisions about planned LPET birth might be reached so as to simultaneously maximize parents' satisfaction with their involvement in the decision‐making process, keep LPET birth to a minimum, and ensure that clinicians are able to fulfill their responsibilities for mothers' and babies' safety. We conducted a two‐part qualitative investigation of the views and experiences of parents and healthcare professionals involved in such decisions, with the aim of identifying areas of commonality and potential tension between these two groups and ways in which these might be addressed. This paper reports the first part of the investigation, in which we explored the input expectant parents want and expect in decision‐making about possible planned LPET birth, how their experiences compare with those wishes and expectations, and how they feel about the input they are able to have.

## 2 Methods

The design of the study was informed by both parents and healthcare professionals and involved semi‐structured interviews with parents of babies up to 6 months old. Parents were included if they were aged 18 or over, had discussed the possibility of planned LPET birth with a doctor in the UK, and had given birth in the UK. Interviews were carried out online or in person by [Researcher A] (Appendix A), recorded, then transcribed by [Researcher A] or [Researcher B]. Recruitment took place via social media, community groups and snowballing from November 2023 to September 2024, when the themes drawn from the data were considered to have sufficient informative power [33] to have met the aims of the study. Written, informed consent was given by all participants (Table 1). Data were pseudonymized prior to analysis.

**TABLE 1:** Participant demographic characteristics.

Characteristic	*N*
Parent's age (years) (missing = 1)	
20–29	2
≥ 30	9
Mother's ethnicity	
White British	8
Indian or Indian British	2
Mixed/multiple ethnicities	2
Previous pregnancies	
0	7
≥ 1	5
Reason early birth was considered (separately or in combination)	
Maternal (obstetric history, older age, hypertension)	3
Fetal (large/small estimated birthweight, slowed growth, reduced movements, fetal distress, polyhydramnios)	11
Baby's gestational stage at birth (weeks^+days^)	
Late preterm (34^+0^–36^+6^)	1
Early term (37^+0^–38^+6^)	4
Full term (≥ 39^+0^)	4

### 2.1 Analysis of Data

Data were analyzed jointly by [Researcher A] and [Researcher B] following Braun & Clarke's six‐phase process for Reflexive Thematic Analysis (RTA) [33, 34, 35], with NVivo qualitative data analysis software as an organizing tool. The analysis was inductive and carried out from a critical realist standpoint, which simultaneously encompasses the ontological belief that an objective reality exists and the epistemological view that truly objective knowledge of social events can never be acquired. This is because both the accounts provided by participants and the interpretations made by researchers are inevitably subjective and shaped by the cultural, linguistic, and other relevant contexts of each. The overarching aim of the analysis was, therefore, to provide a coherent interpretation of the data, grounded in participants' accounts, while also acknowledging the situated nature of the “reality” described within them and the limits and constraints imposed by the worlds of both the participants and the researcher.

In the first phase of the analysis, each researcher individually familiarized themselves with the dataset and produced preliminary mind maps. The researchers worked together on each of the remaining phases, beginning with line‐by‐line scrutiny of transcripts and the development and definition of codes. All data items relevant to the research questions were coded. The coding framework developed iteratively, being repeatedly reviewed and refined. Codes were grouped into broad, overarching themes, each with sub‐themes that related closely to the theme but were distinct from each other. Themes and sub‐themes were checked back against the dataset.

In order to maintain parity across the analysis, where parent‐partner couples were interviewed—either together or individually—their comments were treated separately in the analysis. Differences in perspectives across couples were noted and taken into account when directly relevant to the research questions.

Both researchers trained as nurses and were therefore familiar with the environment and some of the organizational issues that parents were describing. Both have been involved as mothers in decisions about planned early term birth. Their positionality was discussed and reflected upon during data collection and analysis, using reflexive journals to support their reflections.

Various additional measures, appropriate to the design of the study, were taken to strengthen the trustworthiness of the findings. These include thorough documentation of sampling strategies, research procedures, and decisions made during the study; detailed records of decisions made and data analysis processes; and debriefing with peers with appropriate knowledge and skills.

## 3 Results

Twelve parents of nine singleton babies took part in the study. Two mother–father couples were interviewed together and a third in separate interviews. Six further mothers were interviewed alone (Table 1).

**FIGURE 1:**
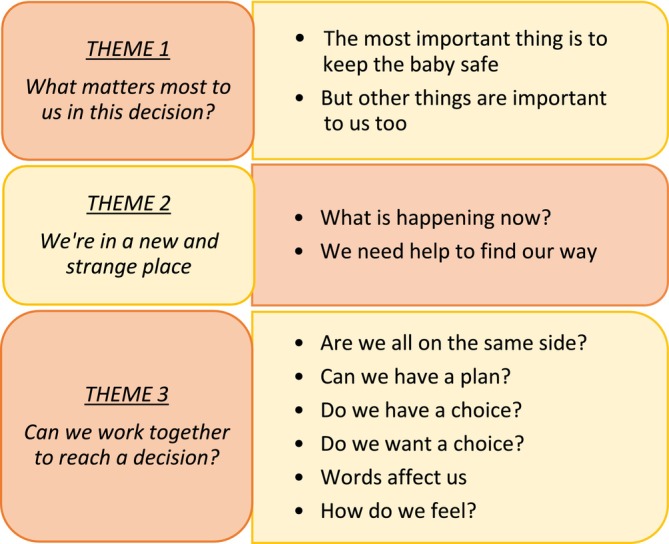
Themes and sub‐themes. [Colour figure can be viewed at wileyonlinelibrary.com]

Three themes and 10 sub‐themes were generated as a result of the analysis (Figure 1). Each was given titles reflecting the parents' standpoint.

### 3.1 Theme 1: What Matters Most to Us in This Decision?

This theme encompasses parents' weighing up their priorities when considering a possible LPET birth. It includes two sub‐themes.

#### 3.1.1 Sub‐Theme 1.1: The Most Important Thing Is to Keep the Baby Safe

The baby's safety was the key motivator for all involved in the decision‐making. Parents were generally accepting of recommendations clearly proposed with safety as the top priority and were reluctant to risk the emotions that would follow rejection of these should adverse outcomes arise.

My husband and I discussed that really the priority was that he came out healthily and safely. (‘Annie’—mother)

I was like, ‘If it's better for him to be out, I'd rather that.’, because I was just really scared that I don't want anything to happen. (‘Jamila’—mother)

It was notable that parents perceived early term babies to be effectively full‐term and viewed by their healthcare professionals as being at no greater risk of adverse consequences if born at that stage:

They did go into their reasoning behind [planned early term birth] but not so much whether there would be any issues because she was early. It was more just, ‘Oh well, she's term, so she can come out.’ (‘Victoria’—mother)

#### 3.1.2 Sub‐Theme 1.2: But Other Things Are Important to Us Too

Where no single option was clearly the best in terms of the baby's safety, parents took into account other issues important to them, such as wanting to experience spontaneous vaginal delivery and how prepared their home was for the baby's arrival:

I really wanted to try and going into […] natural labour, because I didn't have the opportunity with [first baby], so I was trying to hold out for that. (‘Louise’—mother)

I think we were just ‘Right, okay, you're gonna discharge us. We'll be able to go and sort out our house because we've got none of that done […], we're not ready for it’. (‘Max’—father)

While keeping their baby safe was the most important consideration for parents when making decisions on a possible LPET birth, parents reported that other matters of importance to them also influenced their decision.

### 3.2 Theme 2: We Are in a New and Strange Place

This theme, comprising two sub‐themes, relates to the situation where parents need to be involved in decisions about their babies as a result of unanticipated, mostly unprecedented, and sometimes urgent situations, in liaison with people whose roles they did not always know and in a setting that was largely unfamiliar.

#### 3.2.1 Sub‐Theme 2.1: What Is Happening Now?

Parents reported not having fully understood their situation or who was handling a particular aspect of their care:

I was just kind of taking it all in, really, and trying to understand what they were trying to say to me. (‘Aamiina’—mother)

Tell us what needs to be done because […] I haven't got the foggiest as to what's going on… (laughs). (‘Max’—father)

They had a sort of specialist come and give me a scan. (‘Annie’—mother)

Some experienced negative emotional reactions as a result:

They said that if you've not got in touch with us, your induction will be on Monday. So then I was like panicking because I was like, ‘Okay, I might have a baby soon, and I have no idea what's going on. Can't get in touch with anyone. I have no idea what's going on’. (‘Jamila’—mother)

#### 3.2.2 Sub‐Theme 2.2: We Need Help to Find Our Way

This sub‐theme concerns the information parents were given or sought for themselves and the extent to which this met their needs. In some cases, the information received was clear and sufficient:

She explained it really well to us. Like all the different things and even the medical terms. She then took the time to explain what that all meant and the implications […]. (‘Louise’—mother’)

Other parents judged the information they were given as inadequate:

It just felt like I wasn't well informed […] If they explained everything to me and they gave me the pros and cons […] I think that would make me feel a bit like I had more of a reason to what my decision would be. (‘Jamila’—mother)

When asked about the provision of information specific to the increased risks of LPET compared to full term birth, all parents responded that none had been received. Parents varied in their views, even within couples, on whether this information would have been helpful and if so at what stage:

I suppose, they don't want to spook soon‐to‐be parents but […] earlier in the pregnancy say that ‘This is a possibility, this is what it might mean, but here's the information now’. (‘Max’—father)

From the moment I was pregnant, I was anxious. I was terrified something was gonna go wrong. So if I'm then given information about something that may or may not happen […] it's just another level of worry and I don't know (long pause) I don't know if that would have helped me or not. (‘Victoria’—mother)

When faced with the unanticipated need to consider an LPET birth, parents reported that they did not understand what was happening or who they were speaking to. While some parents felt they received clear and sufficient information about the situation, others did not, and no parent received information regarding the increased risks of LPET compared to full term birth.

### 3.3 Theme 3: Can We Work Together to Reach a Decision?

This theme explores how the relationship between parents and healthcare professionals influences the sharing of decision‐making. It incorporates six sub‐themes:

#### 3.3.1 Sub‐Theme 3.1: Are We All on the Same Side?

Some parents described feeling highly supported by their doctors during the decision‐making, but others found their relationships lacking:

The consultant […] gave me her number and she said ‘You could think about what you want to do and you can call up at any point and book your induction in, or whatever you wanna do’. (‘Louise’—mother)

I said to my husband ‘I really want to trust [the consultant], but something's just making me feel like I'm not’. (‘Masarra’—mother)

In other cases, midwives were reported as having advised or advocated on parents' behalf:

The midwife there was saying ‘This isn't right, you shouldn't have had this many […] indications of low movement and then not had any sort of review’. (‘Thomas’—father)

#### 3.3.2 Sub‐Theme 3.2: Can We Have a Plan?

The quality and timing of care planning was a key factor in parents' decision‐making experiences, with almost all mentioning these. For some, the possibility of planned early birth was raised at short notice:

We hadn't realised that 38 weeks was gonna be an option until we were sitting there. Then the obstetrician […] said ‘Well, you could go in today’. (‘Gary’—father)

For others, no plan was formulated despite a request for one:

I said to him, ‘Just in case, can we put a plan in place?’, you know, and he didn't feel like there was a need for it at all. (‘Masarra’—mother)

Parents found the absence of a desired, timely plan difficult to cope with emotionally:

So at that point, I'm stressing out. I'm like, ‘This is ridiculous now […] we've had seven bouts of low movement. She's now not growing. What are we doing about this?’ (‘Rani’—mother)

#### 3.3.3 Sub‐Theme 3.3: Do We Have a Choice?

There were wide differences in the extent of choice parents reported having been given in the decision‐making. Some were given full choice, with or without a specific recommendation:

They did come and discuss it with us and they gave us the option of staying in or going home [overnight] or ‘waiting till the next week’. (‘Victoria’—mother)

Others reported having been given little or no choice:

From pregnancy all the way up to labour, it was very much decisions by the doctors instead of, you know, explaining to me […] ‘Do you want to do this instead […]?’ (‘Aamiina’—mother)

And they said […] they were going to book me in for an induction. (‘Laura’—mother)

Laura reported that her distress at being informed of a decision rather than being involved in it was compounded by it being relayed to her over the telephone, when she was alone, by a consultant she had never met.

#### 3.3.4 Sub‐Theme 3.4: Do We Want a Choice?

Parents who were given options varied in the responsibility they wanted and made use of in the decision‐making process. Some felt insufficiently qualified to choose and would have preferred not to have to do so:

As much as it was nice to have the option, how was it down to people that have only had one baby to decide when this baby comes out? (‘Gary’—father)

Some simply accepted the recommended course of action in deference to obstetricians' knowledge:

You are the experts, I'm gonna put our trust in you. (‘Max’—father)

Older parents and those with more extensive obstetric histories felt better equipped to challenge and ask questions:

[…] because I'm a bit older and perhaps I had been a bit happier to ask more questions or to challenge them a bit more. Whereas, I don't know, perhaps if I had been 25, I might have not. (‘Annie’—mother)

I think because I have experience I felt confident telling them […] what I wasn't okay with, asking them questions. Whereas I think if it was like my first or my second, I'd probably just trust them straight away and be like, okay, let's do that. (‘Nicole’—mother)

Some fathers opted out of the decision‐making, viewing it as more appropriate to defer the choice and responsibility to their partners:

I think the final decision would have always been [wife's], which is how it should be,are in my opinion at least. (‘Thomas’—father)

I was like, ‘I don't know what to do’ and [husband] was like ‘Only you know what is best for you, so do what is best for you’. (‘Jamila’—mother)

#### 3.3.5 Sub‐Theme 3.5: Words Affect Us

The words used by healthcare professionals could have a powerful impact on parents, staying in their memories long after they were spoken. This was particularly evident when parents reported feeling that they were being deprived of choice or that their concerns were dismissed:

All I can remember at that time was the language they were using, kind of ‘We need to induce you.’ or ‘We're gonna do this’ or ‘We're gonna do that’. (‘Aamiina’—mother)

[…] he was just like, oh, you know, ‘Go home. Enjoy it, Be positive’. (‘Masarra’—mother)

I was like, ‘She hasn't got space to move […] this isn't normal for her’. And they were like, ‘Oh, no, no, no, it's not, it's not a thing’. (‘Rani’—mother)

#### 3.3.6 Sub‐Theme 3.6: How Do We Feel?

When asked how they felt during and about the decision‐making process, parents reported a range of emotions, but most involved stress and anxiety over their baby's safety or worries about having to choose a way forward:

I think the fact we got to […] so many bouts of low movement and nothing had ever been mentioned. I think that's what worried me the most […]. (‘Rani—mother’)

I think that's […] why my anxiety started because it was like ‘If I don't do this, what if something bad happens to him. If I do do this and he's born like, when he shouldn't be born, is that also bad for him?’. (‘Jamila’—mother)

This theme relates to the relationship between parents and healthcare professionals. Some parents felt very supported by healthcare professionals, but others did not. Parents were keen to have a plan of action and some reported frustration with a lack of a plan. While some parents wanted to have a choice in decision‐making around a planned LPET birth, others would have preferred not to choose. Parents found the words used by healthcare professionals to have a long‐lasting impact on their emotions. Most frequently, parents reported feelings of stress and anxiety about having to make a decision on an LPET birth.

## 4 Discussion

**FIGURE 2:**
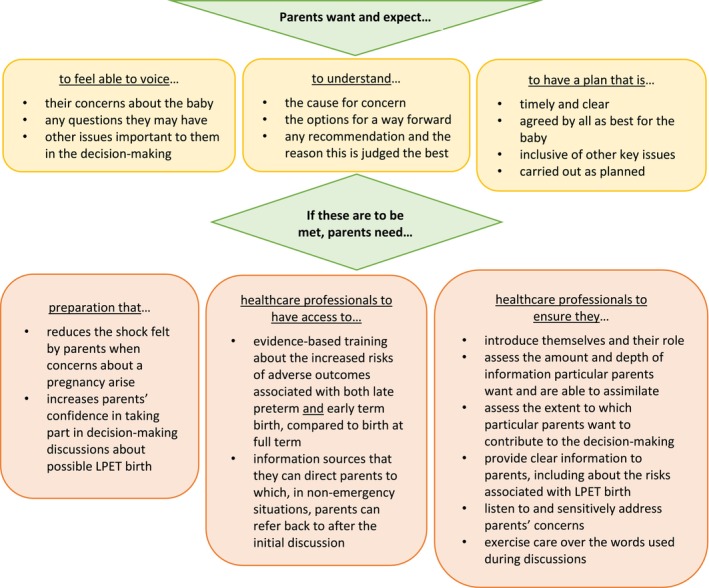
What parents need if their wishes and expectations around decision‐making for possible LPET births are to be met. [Colour figure can be viewed at wileyonlinelibrary.com]

To our knowledge, this is the first qualitative exploration of parents' views and experiences concerning decision‐making about possible planned LPET birth. It has highlighted a complex interplay of influences on their input to the decision‐making process, including their priorities, their understanding of their situation and the maternity care system, and their relationships with the healthcare professionals involved. Parent preparation and training for healthcare professionals have been identified as possible avenues for enhancing understanding and provision of the support needed by parents involved in decision‐making about possible planned LPET birth in the future, in order that their experiences might be more closely aligned with their wishes and expectations (Figure 2).

### 4.1 What the Themes and Sub‐Themes Reveal

While some of the issues highlighted in the themes and sub‐themes generated from the data are applicable to perinatal decision‐making in general, a number were specific to parents involved in discussions about possible planned LPET birth. These included: the disruption to parents' previous hopes and plans in respect of the timing and possibly also the mode of birth; the need to absorb the relative risks of early birth versus continuing a pregnancy in a relatively short time frame; the role of age and previous obstetric experience in enabling parents to feel able to ask questions or challenge a recommendation for LPET birth; and the view of early term birth, held by both parents and healthcare professionals, as being equivalent to full‐term.

Taking both these specific and more general issues into account, we used the themes and sub‐themes generated from the data to inform a model of care for parents involved in decision‐making for a possible LPET birth. Figure 2 summarizes parents' wishes and expectations and proposes what parents need if these wishes and expectations are to be met.

### 4.2 Parent Preparation

The need for adequate preparation for parents in terms of both the possible need for consideration of LPET birth and the increased risks associated with birth at these gestations are both important issues for providers of care. In the UK, community midwives are trained specialists who provide much of the obstetric care for women with a normal pregnancy in community and other out‐of‐hospital settings. Since all parents described their community midwives in highly positive terms, we recommend that work be conducted to explore the potential benefits of two interventions involving the latter: (i) discussing with parents the possibility that complications may arise late in the pregnancy, necessitating discussions about the possible need for LPET birth; and (ii) using their experience of discussing birth timing options with obstetricians to guide parents in how best to make their voices heard during decision‐making discussions.

Recent research by McLeish and Redshaw [34] identified women with multiple disadvantages, defined in terms of having low socio‐economic status and meeting at least one other criterion (such as being a single parent, aged under 25 or a recent migrant) as experiencing confusion and anxiety during their maternity care and lacking confidence in their interactions with those delivering that care. As those who took part in our investigation would not have met McLeish and Redshaw's criteria for low socio‐economic status, the findings reported here have built on what they reported by showing that women who are less disadvantaged but whose pregnancy is not going to plan may experience similar emotions and therefore need additional support. The younger women in the study and those lacking relevant obstetric experience tended to be less confident about asking questions or challenging recommendations, so some form of advanced preparation for these groups in particular could increase their ability to contribute effectively to the decision‐making.

The results of the study highlight that further investigation is needed to explore differences between parents regarding the amount and timing of information they would like about the possibility of LPET birth and associated outcomes. In addition, as the nature of information provision on health‐related issues is evolving (and now includes telephone support, online resources, and apps, as well as the more traditional printed leaflets), exploration is needed of which media would be most helpful and accessible to those requiring information about the reasons for and risks associated with LPET birth.

### 4.3 Training of Healthcare Professionals

Parents' relationships with those providing their care were central to the issues identified in this study, supporting earlier findings identifying the importance of physician empathy and shared responsibility for decision‐making [35, 36, 37, 38]. As is clear from Figure 2, some measures would be straightforward for healthcare professionals to employ, such as always introducing themselves by both name and role; others would necessitate training and/or further research. A key issue that could be addressed by training is the equating of early term with full term birth in terms of the risks of adverse outcomes. While some healthcare professionals do now have an awareness that risks are higher in babies born before 39 weeks of gestation [39], it is important to ensure that both doctors and midwives are aware of the risks associated with LPET birth so that parents can be appropriately informed and relevant action taken. Once awareness of these issues has been raised, it would also be important to provide training in the importance of providing parents with appropriate information, at the time and to the extent they require, on the possible adverse outcomes of LPET birth.

Skill is required to accurately assess the amount and depth of information any parent wants and is able to assimilate, as well as the extent they want to contribute to the decision‐making. As recommended in recent national guidelines [20], these skills could be fostered by training in the principles of shared decision‐making and in the application of models such as the Three‐talk model [40, 41], in which choice is introduced into the conversation, options are described (often with the aid of decision support tools), and people are assisted in exploring their preferences and making decisions.

### 4.4 Strengths and Limitations

A number of participants expressed gratitude for having been given the opportunity to voice and discuss the more negative aspects of their decision‐making experiences, and it is likely that parents who chose to take part in the study represent those less satisfied with the input they were able to have in the decision‐making. Despite efforts to recruit parents from a range of backgrounds, the study is limited by a lack of younger parents, parents expecting more than one baby, those from lower socioeconomic groups, and those of East European and Black ethnicity. However, our sample did include parents who varied in terms of ethnic background, parity, occupation, location of residence in England, and type of housing. These differences between participants contributed to the richness and diversity of the data acquired, which was judged to have provided sufficient informative power for the research question to be addressed.

## 5 Conclusions

This study has added to the existing literature on obstetric decision‐making, both generally and specific to LPET birth, by identifying parents' needs to be able to make their voices heard, to understand their situation and options, and to have a plan acceptable to all involved. It has signposted some clear directions for parental support, as well as for healthcare professionals' training and practice, in order that parents' decision‐making experiences be optimized. The study has also identified areas where further research is needed, particularly with respect to parent preparation for discussions about possible LPET birth, the timing of information provision, and the media by which it would be most helpfully provided.

## Ethics Statement

The study was approved by the University of Leicester's Medical and Biological Sciences Research Ethics Committee in October 2023.

## Conflicts of Interest

The authors declare no conflicts of interest.

## Data Availability

The data that support the findings of this study are available from the corresponding author upon reasonable request.

## Appendix A Interview Topic Guide for Parents Taking Part in the PIP Study

### Introductory Questions

- Before we start the main part of the interview, can you please tell me a little bit about yourself and your family. *Prompts*:
  ○ *How many children do you have (age/s, name/s)?*
  ○ *Who do you live with?*
  ○ *What sort of property (house, flat, etc.)?*
  ○ *Job (self, partner, FT, PT)?*
- [*If* > *1 child*] Which of your children did the discussion about possible early birth concern?
- What was the decision?
- How did things go? *Prompt*:
  ○ *Did the birth go as planned? How was the baby afterwards?*

### Key Questions

- Thinking back to when you first found out that your baby might need to be born early, how did that make you feel?
- Can you tell me a bit about the first discussion about that? *Prompts*:
  ○ *who took part and when/where did it take place?*
  ○ *what information was provided (the reasons early birth was being considered/any possible alternatives/pros and cons of each)?*
  ○ *recommendation/reason?*
- How involved did you feel in the discussion? *Prompt*:
  ○ *what made you feel this way?*
- Were you given some time to think about things and have a further discussion before the decision was made [for/against early birth]? *Prompt*:
  ○ *If not, was this because there was a medical reason for your baby to be born straight away?*
- Did you discuss the situation with anyone else before the decision was made? *Prompts*:
  ○ *who (spouse/partner, own parent, friend, other HCP)?*
  ○ *where and when did the discussions take place?*
- Was the decision for your baby [to be/not to be] born early made in the way you had expected?
- How did you feel at the time about the way it was made?
- How do you feel about that now?
- Would you like anything to have been done or said differently?
- Was there anything particularly good about the decision‐making?

### Final Questions

- If you had the chance to talk to the people who were involved in your care, is there anything you would like to say to them?
- Is there anything else you'd like to add before we end the interview?

## References

[1] Office for National Statistics , Birth Characteristics (ONS), accessed June 3, 2025, www.ons.gov.uk/peoplepopulationandcommunity/birthsdeathsandmarriages/livebirths/datasets/birthcharacteristicsinenglandandwales.

[2] S. Sengupta , V. Carrion , J. Shelton , et al., “Adverse Neonatal Outcomes Associated With Early‐Term Birth,” JAMA Pediatrics 167, no. 11 (2013): 1053–1059, 10.1001/jamapediatrics.2013.2581. 24080985

[3] E. M. Boyle , S. Johnson , B. Manktelow , et al., “Neonatal Outcomes and Delivery of Care for Infants Born Late Preterm or Moderately Preterm: A Prospective Population‐Based Study,” Archives of Disease in Childhood‐Fetal and Neonatal Edition 100, no. 6 (2015): 479, 10.1136/archdischild-2014-307347. PMC4680176 25834169

[4] C. J. Chantry , K. G. Dewey , J. M. Peerson , E. A. Wagner , and L. Nommsen‐Rivers , “In‐Hospital Formula Use Increases Early Breastfeeding Cessation Among First‐Time Mothers Intending to Exclusively Breastfeed,” Journal of Pediatrics 164, no. 6 (2014): 1339–1345.e5, 10.1016/j.jpeds.2013.12.035. 24529621 PMC4120190

[5] T. Muganthan and E. Boyle , “Early Childhood Health and Morbidity, Including Respiratory Function in Late Preterm and Early Term Births,” Seminars in Fetal and Neonatal Medicine 24 (2019): 48–53. 30348617 10.1016/j.siny.2018.10.007

[6] I. Ferreira , P. T. Gbatu , and C. A. Boreham , “Gestational Age and Cardiorespiratory Fitness in Individuals Born at Term: A Life Course Study,” Journal of the American Heart Association 6, no. 10 (2017), 10.1161/JAHA.117.006467. PMC5721855 28954725

[7] E. Kajantie , S. Strang‐Karlsson , K. A. I. Evensen , and P. Haaramo , “Adult Outcomes of Being Born Late Preterm or Early Term—What Do We Know?,” Seminars in Fetal and Neonatal Medicine 24 (2019): 66–83. 30420114 10.1016/j.siny.2018.11.001

[8] S. Johnson , T. A. Evans , E. S. Draper , et al., “Neurodevelopmental Outcomes Following Late and Moderate Prematurity: A Population‐Based Cohort Study,” Archives of Disease in Childhood‐Fetal and Neonatal Edition 100, no. 4 (2015): F301–F308, 10.1136/archdischild-2014-307684. 25834170 PMC4484499

[9] S. Johnson , R. J. Matthews , E. S. Draper , et al., “Early Emergence of Delayed Social Competence in Infants Born Late and Moderately Preterm,” Journal of Developmental and Behavioral Pediatrics 36, no. 9 (2015): 690–699, 10.1097/DBP.0000000000000222. 26461097

[10] C. Crump , K. Sundquist , J. Sundquist , and M. A. Winkleby , “Gestational Age at Birth and Mortality in Young Adulthood,” Journal of the American Medical Association 306, no. 11 (2011): 1233–1240, 10.1001/jama.2011.1331. 21934056

[11] T. Isayama , A. Lewis‐Mikhael , D. O'Reilly , J. Beyene , and S. D. McDonald , “Health Services Use by Late Preterm and Term Infants From Infancy to Adulthood: A Meta‐Analysis,” Pediatrics 140, no. 1 (2017): e20170266, 10.1542/peds.2017-0266. 28759410

[12] K. Heinonen , J. G. Eriksson , J. Lahti , et al., “Late Preterm Birth and Neurocognitive Performance in Late Adulthood: A Birth Cohort Study,” Pediatrics 135, no. 4 (2015): 818, 10.1542/peds.2014-3556. 25733746

[13] K. A. Khan , S. Petrou , M. Dritsaki , et al., “Economic Costs Associated With Moderate and Late Preterm Birth: A Prospective Population‐Based Study,” BJOG: An International Journal of Obstetrics and Gynaecology 122, no. 11 (2015): 1495–1505, 10.1111/1471-0528.13515. 26219352

[14] E. R. Cheng , H. McGough , and B. Tucker Edmonds , “Paternal Preferences, Perspectives, and Involvement in Perinatal Decision Making,” Obstetrical and Gynecological Survey 74, no. 3 (2019): 170–177, 10.1097/ogx.0000000000000650. 31634920

[15] D. Coates , N. Donnolley , M. Foureur , P. Thirukumar , and A. Henry , “Factors Associated With Women's Birth Beliefs and Experiences of Decision‐Making in the Context of Planned Birth: A Survey Study,” Midwifery 96 (2021): 102944, 10.1016/j.midw.2021.102944. 33610064

[16] C. Dupont , P. Blanc‐Petitjean , M. Cortet , et al., “Dissatisfaction of Women With Induction of Labour According to Parity: Results of a Population‐Based Cohort Study,” Midwifery 84 (2020): 102663, 10.1016/j.midw.2020.102663. 32092607

[17] B. Tucker Edmonds , T. A. Savage , R. E. Kimura , et al., “Prospective Parents' Perspectives on Antenatal Decision Making for the Anticipated Birth of a Periviable Infant,” Journal of Maternal‐Fetal and Neonatal Medicine 32, no. 5 (2019): 820–825, 10.1080/14767058.2017.1393066. 29103318 PMC6810652

[18] C. Dageville , P. Bétrémieux , F. Gold , and U. Simeoni , “The French Society of Neonatology's Proposals for Neonatal End‐of‐Life Decision‐Making,” Neonatology 100, no. 2 (2011): 206–214, 10.1159/000324119. 21471705

[19] R. Geurtzen , A. F. J. van Heijst , J. M. T. Draaisma , et al., “Development of Nationwide Recommendations to Support Prenatal Counseling in Extreme Prematurity,” Pediatrics 143, no. 6 (2019): e20183253, 10.1542/peds.2018-3253. 31160512

[20] National Institute for Health and Care Excellence (NICE) , Shared Decision Making. NICE Guideline [NG 197] (NICE, 2021). 34339147

[21] National Institute for Health and Care Excellence (NICE) , Caesarean Birth. NICE Guideline [NG 192] (NICE, 2021). 33877751

[22] T. M. Berger , V. Bernet , S. El Alama , et al., “Perinatal Care at the Limit of Viability Between 22 and 26 Completed Weeks of Gestation in Switzerland: 2011 Revision of the Swiss Recommendations,” Swiss Medical Weekly 141 (2011): w13280, 10.4414/smw.2011.13280. 22009720

[23] B. Kirkup , Reading the Signals: Maternity and Neonatal Services in East Kent—The Report of the Independent Investigation (2022), https://assets.publishing.service.gov.uk/government/uploads/system/uploads/attachment_data/file/1111992/reading‐the‐signals‐maternity‐and‐neonatal‐services‐in‐east‐kent_the‐report‐of‐the‐independent‐investigation_print‐ready.pdf.

[24] D. Ockenden , Findings, Conclusions and Essential Actions From the Independent Review of Maternity Services at The Shrewsbury and Telford Hospital NHS Trust: Our Final Report (2022), https://www.ockendenmaternityreview.org.uk/wp‐content/uploads/2022/03/FINAL_INDEPENDENT_MATERNITY_REVIEW_OF_MATERNITY_SERVICES_REPORT.pdf.

[25] M. K. Longworth , C. Furber , and S. Kirk , “A Narrative Review of Fathers' Involvement During Labour and Birth and Their Influence on Decision Making,” Midwifery 31, no. 9 (2015): 844–857, 10.1016/j.midw.2015.06.004. 26165171

[26] M. Danerek , K. Maršál , M. Cuttini , G. Lingman , T. Nilstun , and A. K. Dykes , “Attitudes of Midwives in Sweden Toward a Woman's Refusal of an Emergency Cesarean Section or a Cesarean Section on Request,” Birth: Issues in Perinatal Care 38, no. 1 (2011): 71–79, 10.1111/j.1523-536X.2010.00440.x. 21332777

[27] K. T. Eide and K. Bærøe , “How to Reach Trustworthy Decisions for Caesarean Sections on Maternal Request: A Call for Beneficial Power,” Journal of Medical Ethics 47 (2020): e45, 10.1136/medethics-2020-106071. 33055135 PMC8639926

[28] D. G. Batton , “Clinical Report—Antenatal Counseling Regarding Resuscitation at an Extremely Low Gestational Age,” Pediatrics 124, no. 1 (2009): 422–427, 10.1542/peds.2009-1060. 19564329

[29] B. T. Edmonds , F. McKenzie , K. S. Hendrix , S. M. Perkins , and G. D. Zimet , “The Influence of Resuscitation Preferences on Obstetrical Management of Periviable Deliveries,” Journal of Perinatology 35, no. 3 (2015): 161–166, 10.1038/jp.2014.175. 25254331 PMC4414321

[30] M. Dugas , A. Shorten , E. Dubé , M. Wassef , E. Bujold , and N. Chaillet , “Decision Aid Tools to Support Women's Decision Making in Pregnancy and Birth: A Systematic Review and Meta‐Analysis,” Social Science & Medicine 74, no. 12 (2012): 1968–1978, 10.1016/j.socscimed.2012.01.041. 22475401

[31] J. Henderson , H. Gao , and M. Redshaw , “Experiencing Maternity Care: The Care Received and Perceptions of Women From Different Ethnic Groups,” BMC Pregnancy and Childbirth 13 (2013): 196, 10.1186/1471-2393-13-196. 24148317 PMC3854085

[32] F. J. Mielewczyk and E. M. Boyle , “Uncharted Territory: A Narrative Review of Parental Involvement in Decision‐Making About Late Preterm and Early Term Delivery,” BMC Pregnancy and Childbirth 23, no. 1 (2023): 526, 10.1186/s12884-023-05845-6. 37464284 PMC10354979

[33] V. Braun and V. Clarke , Thematic Analysis: A Practical Guide (Sage, 2022), 338.

[34] J. McLeish and M. Redshaw , “Maternity Experiences of Mothers With Multiple Disadvantages in England: A Qualitative Study,” Women and Birth 32, no. 2 (2019): 178–184, 10.1016/j.wombi.2018.05.009. 29910026 PMC7074001

[35] A. Paton , N. Armstrong , L. Smith , and R. Lotto , “Parents' Decision‐Making Following Diagnosis of a Severe Congenital Anomaly in Pregnancy: Practical, Theoretical and Ethical Tensions,” Social Science & Medicine 266 (2020): 113362, 10.1016/j.socscimed.2020.113362. 32957025

[36] X. Zhang , L. Li , Q. Zhang , L. H. Le , and Y. Wu , “Physician Empathy in Doctor‐Patient Communication: A Systematic Review,” Health Communication 39, no. 5 (2024): 1027–1037, 10.1080/10410236.2023.2201735. 37062918

[37] H. K. Pellikka , A. Axelin , U. Sankilampi , and M. Kangasniemi , “Shared Responsibility for Decision‐Making in NICU: A Scoping Review,” Nursing Ethics 30, no. 3 (2023): 462–476, 10.1177/09697330221134948. 36688269 PMC10185855

[38] S. W. Chan , E. Tulloch , E. S. Cooper , A. Smith , W. Wojcik , and J. E. Norman , “Montgomery and Informed Consent: Where Are We Now?,” BMJ 357 (2017): j2224, 10.1136/bmj.j2224. 28500035

[39] F. J. Mielewczyk , C. A. Mulvaney , and E. M. Boyle , Parental Involvement in Decision‐Making About Planned Late Preterm and Early Term Birth (the ‘PIP’ Study): Part 2—A Reflexive Thematic Analysis of Interviews With Obstetricians, Neonatologists and Midwives. Unpublished Work. 10.1111/birt.70025 PMC13156663 41170859

[40] G. Elwyn , M. A. Durand , J. Song , et al., “A Three‐Talk Model for Shared Decision Making: Multistage Consultation Process,” BMJ 359 (2017): j4891, 10.1136/bmj.j4891. 29109079 PMC5683042

[41] G. Elwyn , D. Frosch , R. Thomson , et al., “Shared Decision Making: A Model for Clinical Practice,” Journal of General Internal Medicine 27, no. 10 (2012): 1361–1367, 10.1007/s11606-012-2077-6. 22618581 PMC3445676

